# A practical guide to public involvement with children and young people in dental research

**DOI:** 10.1038/s41415-024-7936-0

**Published:** 2025-01-24

**Authors:** Emma Morgan, Jennifer Preston, Sondos Albadri

**Affiliations:** 41415261075001 NIHR Academic Clinical Fellow in Paediatric Dentistry, University of Liverpool, Liverpool, UK; 41415261075002 Patient and Public Involvement Policy Manager, Faculty of Health and Life Sciences, University of Liverpool, Liverpool, UK; 41415261075003 Professor in Paediatric Dentistry, University of Liverpool, Liverpool, UK

## Abstract

Public involvement (PI) in health research is an umbrella term which describes the process by which research is undertaken ‘with' or ‘by' people rather than ‘to', ‘about' or ‘for' them. The United Nations Convention of the Rights of the Child provides children and young people (CYP) with a comprehensive set of human rights. In line with Article 12, every child has a right to express their views in all matters which may affect them. Additionally, there has been increased expectation from funders for PI to be demonstrated as part of research. While PI encompasses all activities which aim to involve CYP, they can be categorised into different levels, including consultation, collaboration and user-led. CYP can be involved in many different aspects of research, from research question identification, research design and dissemination. Despite this, there may be challenges to delivering PI, such as funding and time. Using the basic principles outlined in this paper, there is opportunity for involvement of CYP in a range of settings to produce meaningful involvement with CYP.

## Key points

- In line with the United Nations Convention of the Rights of the Child, children and young people have the right to be involved in all matters affecting them.
- Public involvement is a key part of the research process and has benefits for children and young people, the researchers and research outputs.
- This paper provides practical tips to support the delivery of meaningful public involvement.

## What is public involvement?

Public involvement (PI) in health research is an umbrella term which describes the process by which research is undertaken ‘with' or ‘by' people rather than ‘to', ‘about' or ‘for' them.^1^ The terms patient and public involvement (PPI) and PI are often used interchangeably but have subtle differences in their definition. PPI provides separate definitions of patients and the public; patients are seen as current or former users of health and social care services, with the public seen as anybody else, such as potential users of healthcare services. PI encompasses both, including current, former or potential patients and those who represent patients, carers and family members.^1^

Public involvement differs to public engagement. Engagement focuses on the dissemination of research information and knowledge to the public, for example raising awareness of research or disseminating research findings.^1^ PI involves a partnership between the researchers and the public, empowering the public to influence decision-making at all stages of the research process. This may include a range of activities, including prioritising research themes, working as part of a project advisory group, informing the development of research materials, or carrying out user-led research.^1^ Regardless of the activity or stage, PI should be meaningful, empowering the public to inform research development, and not simply a tick-box exercise. It is also important to consider that children and young people (CYP) may also be current, former or potential service users, carers or family members and should be involved in PI.

Health research should have the overarching aim of meeting the needs of the public, including where those groups are CYP. To meet this aim, it is important to work with those who have relevant lived experience or knowledge, including CYP, facilitating their voice to produce research which is relevant to their needs. In recent years, the expectation from funders for PI to be part of the research has increased, with many funders stipulating that applicants demonstrate how the public will actively be involved in the design and delivery of their research.^1^^,^^2^ In 2022, several funding bodies, including UK Research and Innovation and the National Institute for Health and Social Care Research (NIHR) signed a shared commitment to improve public involvement in research, stating that ‘public involvement is important, expected and possible in all types of health and social care research'.^2^ In addition to this, there has been a drive for PI in wider fields, such as NHS service delivery, clinical guideline development and the UK parliamentary system.^3^^,^^4^^,^^5^

**Fig. 1:**
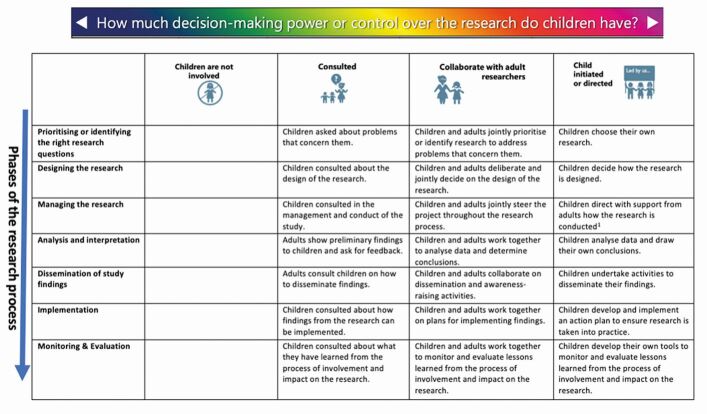
Participation matrix. Reproduced from Preston *et al*., ‘Reporting involvement activities with children and young people in paediatric research: a framework analysis', *Res Involve Engage*, Vol 9, Biomed Central, 2023, licensed under http://creativecommons.org/licenses/by/4.0/6,7

PI encompasses all activities which aim to include the public, including CYP, in the research process; however, the extent to which people are involved in the research process may differ. Involvement with CYP may take place at different phases of the research process and at different levels. Figure 1 highlights an adapted participation matrix, originally developed by Shier, which describes three levels of involvement - consultation, collaboration and user-led - across the different phases of the research process.^6^^,^^7^ Consultation describes a one-off involvement process, where CYP provide opinions on certain aspects of a proposal to inform the research but are not actively involved in decision-making on an ongoing basis.^6^^,^^7^ Collaboration describes ongoing involvement with CYP, where they are actively involved in the research process.^6^^,^^7^ In this case, CYP work alongside researchers providing input into areas such as research design and/or data collection or analysis and/or dissemination. User-led describes a research process which is led by CYP, rather than the researcher.^6^^,^^7^ With support from researchers, CYP design and deliver the research project. This may be the sole study, or there may be PI-led elements within a larger research study.

To undertake high-quality research with CYP, it is important that they are involved in PI and also as participants in the research. It is important to include CYP as active participants in research where they are allowed to provide their experiences and opinions, rather than using proxies, such as parents. In a systematic review published in 2015, only 17.4% of dental research was undertaken with CYP where CYP were participants in the study. Additionally, 18.1% of studies used proxies for CYP and 64.2% undertook research on children, where they were subjects and not involved in the research.^8^ While this is an improvement from 2007, where only 7.3% of dental research was with CYP, there is still a need for significant improvement in the extent to which CYP are involved in research.^9^

## Why involve children and young people?

The United Nations Convention of the Rights of the Child (UNCRC) provides CYP with a comprehensive set of human rights.^10^ Article 12 of the UNCRC states that ‘every child has the right to express their views, feelings, and wishes in all matters affecting them, and to have their views considered and taken seriously'.^11^ CYP should have the opportunity to contribute directly and this input can have many benefits to both CYP and the research.

CYP can be involved from the start, aiding in identification and prioritisation of research questions. Through the design, CYP can inform recruitment strategies, techniques for data collection and dissemination of results.^12^ This can have great benefits for research, including wider involvement of CYP and improved recruitment and retention. Involvement in research supports CYP to develop a wide range of research skills, such as writing and public speaking.^13^ This has been associated with a self-perceived improvement in confidence, self-esteem and employment opportunities.^12^ CYP report positive experiences of involvement in research, such as feeling part of a team, feeling listened too, empowerment and a greater understanding of their rights.^12^

## How to involve CYP into research

Early involvement of CYP may identify a research question relevant to your local community or context, which may not otherwise have been identified. While advocating for early involvement in the research design process, we note this may not be possible, for example, where funding is provided from a pre-defined research question. Despite this, engagement with CYP as early as possible is beneficial for the research and CYP.

It is important to consider the level of involvement you anticipate using: consultation, collaboration, or user-led. There are many factors which may influence this decision, such as time availability, funding availability, type of research being undertaken and previous PI experience. Time and funding availability are some of the biggest limiting factors when considering public involvement, which can impact a researcher's ability to undertake meaningful PI.^12^ Where possible, appropriate time and funding should be incorporated into research design to facilitate ongoing PI. Where this is not possible, a pragmatic approach is needed to consider how CYP can be involved in the research. In these cases, consultation approaches are often used to gain feedback from CYP; however, it vital that this is appropriately planned and the feedback actioned to avoid this moving to a tick-box approach to PI. The nature of research can influence the type of PI planned and, in some studies, it may not be appropriate to have CYP involved in all aspects of the research. However, in such studies, CYP can have a vital role in developing techniques for disseminating research.

Undertaking PI for the first time can be a daunting but it doesn't mean that you can't undertake meaningful PI. While the levels of PI are often described in isolation, there may be a natural development from consultation to collaboration or user-led research. We note that collaboration and user-led research can be easier once the research has developed a relationship with a community of CYP with an interest in this area. Meaningful consultation can have great benefit to research and can help foster partnerships with CYP, opening the door for further involvement where CYP have greater autonomy.

## Where do you start?

PI is a fluid and ever-evolving process and is highly dependent on the research area and the CYP involved. Considering the needs of CYP, it is almost impossible to create a one-size-fits-all approach to PI. However, there is guidance available from a range of sources:

- UNCRC Article 12: the right of the child to be heard^14^
- UK Standards for Public Involvement in research^15^
- NIHR: briefing notes for public involvement in the NHS, health and social research^1^
- Top tips for involving CYP in research from CYP's point of view^16^
- Royal College of Paediatrics and Child Health: engaging children and young people^17^

While ethical approval is often not needed, the underlying ethical principles should still apply to PI processes. These include areas such as informed consent, safeguarding, ensuring confidentiality, minimising risk of harm and training for researchers and PI members (where required).^18^ Ethical approval may be required, for example, for user-led research; although, this remains a point of discussion as outlined by Nollett *et al*.^19^ If unsure, it is important to discuss this with your local institution.

## Who to involve

The CYP involved in PI will depend on the type of research being undertaken and the nature of the input required. It is important to be flexible in your approach in identifying those to be involved in your PI, as this may evolve as your research progresses. Firstly, consider the population you plan to be involved in the research. This may be associated with characteristics such as age, location or a certain health condition or lived experience. Secondly, consider the level of involvement you are looking for, for example, a one-off consultation or a long-term, user-led research project, as this may alter the initial approach. Once the target group has been identified, methods to advertise the involvement opportunities should be considered. It is useful to consider whether your organisation, such as an NHS trust or university, already has an established link to existing groups which can be used. These may include:

- Existing local/regional/national young person's advisory group (YPAG) - identify whether there is a local YPAG in your region. There may be a GenerationR YPAG near you, which is an alliance of YPAGs across the UK funded by NIHR and/or NHS organisations through various channels.^20^ Contact the co-ordinator to discuss involvement of the YPAG
- Existing PI groups relevant to your research theme - there may be a regional or national PI group relevant to your research area. They may be able to be involved in your work or may be able to provide input as to the best place to advertise for the CYP you are looking to involve
- Charities or support groups - a wide range of charity groups, support groups or patient networks exist locally, regionally, nationally and internationally. These groups may be aimed at certain populations, such as those with specific conditions or of a certain age, so it is important to identify if there is a group relevant to your research. These may be of benefit for research regarding rare diseases, as it can help identify those who may be current patients, carers or family members
- Relevant settings - there may be settings which may be best suited to the CYP you are looking to involve. Examples include healthcare settings, activity groups or clubs. It can be useful to contact these areas and discuss the possibility of advertising through these networks. When considering healthcare settings, there may be wider ethical considerations associated with these settings
- Social media - social media may be useful to disseminate this information through wider networks.

The characteristics of those involved also needs to be considered, with a desire to maximise the diversity of the group. There may be groups of CYP who are less likely to be involved in research, and while there is a wide range of terminology used to describe these groups, they are often defined as under-served groups.^21^ This definition best reflects that research should better serve these groups and facilitate their involvement.^21^ Intention must be made to plan ways to actively offer these groups an opportunity to be involved in research. Researchers may consider contacting those who may have an existing relationship with these groups, who can be described as ‘gatekeepers'.^22^ These gatekeepers can be a range of people who work in different settings, such as healthcare professionals in the community, those in community groups, such as children's centres and clubs, or religious groups. Contacting such gatekeepers and explaining the rationale behind the research and the expectations of the PI will be useful. Gatekeepers may suggest adaptions to the planned PI to support CYP involvement and can suggest the best way to advertise to increase involvement. Additionally, advertisement through these gatekeepers, who are often a trusted person within the community, can facilitate rapport building and subsequent involvement, rather than advertising coming from an unknown researcher. Building trust and rapport with gatekeepers and communities takes time and this should be considered in the research planning.

## Practical delivery of PI

Adapting the setting of PI can be useful to help people get CYP involved. Holding events in a location which is familiar to the public can be useful to aid involvement. For example, rather than inviting people to attend a meeting with you at a different location, try to hold a session in a convenient location, or attend a scheduled meeting with an existing group. This can ease the process of involvement and help reduce the burden for CYP.

There are many methods you can use to involve CYP in research. Common examples include questionnaires, interview or focus group discussions, and interactive workshops. The methods used are flexible depending on the CYP involved but they should be encouraging and easy for CYP to give their opinions. Reasonable adjustments should be made to facilitate involvement of CYP who may need additional support for communication. While the input is coming from CYP, some CYP may prefer to have their parent or guardian present for support, while others may not. Discuss this with the CYP involved and make adaptions so that all CYP are comfortable. If parents are present, ensure they have information regarding their role in support to ensure that the CYP's voice leads the discussion. There may need to be several events where CYP of a similar age range are together so that discussions can be pitched at the appropriate level of understanding.

When involving CYP, timing is also of particular importance. CYP often have busy lives, including extra-curricular activities, crucial timings, such as GCSE and A-level exams, and other personal responsibilities. It is important to be flexible, offering after-school times, weekends or school holidays, depending on their preference. Virtual events may be more convenient and can be useful for PI which is required over a large geographical area. Face-to-face events can allow CYP to discuss with each other, but it is important to hold these in a place convenient to the CYP.

It is important to cost sufficient funding for PI. Guidance for remuneration is available from NIHR.^23^ Consider the length of time and level of commitment required for the involvement and be transparent with CYP regarding the commitment. Remuneration should be reflective of the level of commitment and any associated costs, such as travel expenses. Shopping vouchers are a popular method of remuneration for CYP. Direct monetary payment can be considered, such as for travel expenses; although, this can have complexities and local guidance should be followed. Some CYP will want to be involved in research and prefer not to be remunerated for this and it is important to respect these wishes.

## Evaluation of PI

Evaluation is a key component of PI. It is important to consider the impact involvement has on both the public and the research. Firstly, it is important that CYP are provided with feedback regarding the input they have provided and how this influenced the research. It is important that this feedback is transparent and timely. Failure to do so can leave those involved dissatisfied and with feelings of it being a tick-box exercise.^12^ There are many ways that impacts of PI can be shared, such as a newsletter or a website. It is beneficial to discuss the preferred ways of receiving updates with those involved to ensure it is timely and relevant to their needs.

The research team should gain feedback from those involved regarding their experience of the PI process and how it met their expectations. Key areas to evaluate include setting, timing, activities, feeling heard, meeting expectations and areas for development. This can take many formats but questionnaires or open discussions with those involved are commonly used. Reflections from the researcher using a diary can also be helpful to note key discussions or developments from PI.

## Reporting PI

Sharing learning about CYP involvement in practice is aided through systematic evaluation and reporting of what works best for CYP and describes what impact their involvement has on the actual research and on those who get involved. To aid the reporting process, the Guidance for Reporting Involvement of Patients and the Public (GRIPP2) checklist has been developed to improve the quality and consistency of reporting.^24^ An example GRIPP2 short form can be seen in Table 1, outlining the key areas for reporting.

**Table 1:** GRIPP2 reporting checklist^24^

Section and topic	Item
Aim	Report the aim of PI in the study
	In this example, there was scope for further research related to oral health in children with long-term health conditions (LTHC). The research aim had not been identified so PI was required to explore this area and identify a research need.
Methods	Provide a clear description of the methods used for PI in the study
	Advertising: Local support groups and charities for children with specific health conditions were contacted with a request to share an advert for involvement. Where local support groups didn't exist, national groups were contacted to support with advertisement. Additionally, an existing PI group for CYP with LTHC was identified locally and were able to facilitate an event.
Study results	Outcomes - report the results of PI in the study, including both positive and negative outcomes
	Due to the widespread advertising, several small virtual events were held with CYP aged 8-16 years using Zoom and Microsoft Teams. Events consisted of discussions, both verbal or written using the chat function for those who preferred not to speak. Interactive discussion boards were used for CYP to share text and images through the discussions. CYP discussed a range of experiences and identified a need for further research to understand oral health experiences in children with LTHC. This informed the development of a qualitative study in this area utilising semi-structured interviews.
Discussion and conclusions	Outcomes - comment on the extent to which PI influenced the study overall. Describe positive and negative effects
	Undertaking PI early is useful to help explore or identify a potential research question which may not have otherwise been identified. Reduced engagement was noted around school holiday times; however, the timing of this was restricted by funding. Group events can be positive to allow CYP to discuss with others with the same experience. One-to-one events were used utilised where CYP preferred single events or were unable to attend group events due to other commitment. This flexibility helped increase overall engagement. Events with CYP were held separate to parent events. Involving CYP with or without their parents, depends on the topic and preferences of the CYP involved.
Reflections/critical perspective	Comment critically on the study, reflecting on the things that went well and those that did not, so others can learn from this experience
	Make a log as you continue of things which went well and things which could be improved in future as this will help reflection and development. Get feedback from those involved to support this. Initial consultant events have led to collaboration, as those involved expressed an interest in being involved in this going forwards. This demonstrates how consultation can evolve into collaboration or user-led research.

In addition to reporting PI within scientific publications, it is important to consider dissemination of the overall research outputs and PI contributions to the public, relating public involvement to public engagement. There are many methods of public engagement, such as open days or community events, social media and websites, and this will likely vary depending on the nature of the research. This can be beneficial to both demonstrate the impact of PI and research and may encourage others to be involved in research in the future.

This forms part of public engagement, which focuses on the dissemination of research to the public.

## Discussion and conclusion

As demonstrated, there is great scope for PI in dental research, with benefits for the researcher, CYP and the research output. At all levels of PI, there is opportunity for meaningful relationships to be built with CYP to create research which is both achievable and relevant to their needs and desires. While PI can have great benefits to dental research, it is important to acknowledge challenges that research teams face during the PI process. Limitations in time, funding or ability to engage sufficient numbers of CYP are often reported by researchers. However, this does not mean that the PI produced will not be meaningful. Proactive engagement of CYP through a range of methods, transparent reporting and research reflection are key in preventing PI becoming a tick-box exercise. Using these principles, there is opportunity for involvement of CYP in a range of settings and the authors actively encourage readers to involve CYP in decision-making for what is, after all, their research.
